# Improved Transformer for Time Series Senescence Root Recognition

**DOI:** 10.34133/plantphenomics.0159

**Published:** 2024-03-28

**Authors:** Hui Tang, Xue Cheng, Qiushi Yu, JiaXi Zhang, Nan Wang, Liantao Liu

**Affiliations:** ^1^ College of Mechanical and Electrical Engineering, Hebei Agricultural University, 071000 Baoding, China.; ^2^ State Key Laboratory of North China Crop Improvement and Regulation, Hebei Agricultural University, 071000 Baoding, China.; ^3^ College of Agronomy, Hebei Agricultural University, 071000 Baoding, China.

## Abstract

The root is an important organ for plants to obtain nutrients and water, and its phenotypic characteristics are closely related to its functions. Deep-learning-based high-throughput in situ root senescence feature extraction has not yet been published. In light of this, this paper suggests a technique based on the transformer neural network for retrieving cotton’s in situ root senescence properties. High-resolution in situ root pictures with various levels of senescence are the main subject of the investigation. By comparing the semantic segmentation of the root system by general convolutional neural networks and transformer neural networks, SegFormer-UN (large) achieves the optimal evaluation metrics with mIoU, mRecall, mPrecision, and mF1 metric values of 81.52%, 86.87%, 90.98%, and 88.81%, respectively. The segmentation results indicate more accurate predictions at the connections of root systems in the segmented images. In contrast to 2 algorithms for cotton root senescence extraction based on deep learning and image processing, the in situ root senescence recognition algorithm using the SegFormer-UN model has a parameter count of 5.81 million and operates at a fast speed, approximately 4 min per image. It can accurately identify senescence roots in the image. We propose that the SegFormer-UN model can rapidly and nondestructively identify senescence root in in situ root images, providing important methodological support for efficient crop senescence research.

## Introduction

The root is composed of fine root and their root hairs, which are the key tissues that absorb soil nutrients and water, directly affecting the functions of the root [1]. The root is in direct contact with the soil, and because of this, its features adapt to environmental changes before those of aboveground plants, giving a clear indication of how crops are growing [2,3]. Root senescence is an important root trait that importantly affects the process of aboveground senescence [4]. In addition, exploring the law of root senescence is an important aspect of revealing the senescence of aboveground plants.

The basis for root senescence research is the acquisition of dynamic in situ root pictures. Finding and identifying roots are challenging because of the soil’s shade and lack of transparency. Conventional manual excavation techniques including digging, soil coring, and soil column methods [5–7] cannot be used to research the senescence law of roots. The in situ cultivation method cultivates plants while allowing for the observation of root characteristics in an undisturbed environment [8]. In recent years, the commonly used in situ cultivation methods include aeroponics, hydroponics, gel culture, and germination paper culture [9–12]. These techniques have some usefulness for researching in situ roots, but using soil as a culture medium is more challenging, and the quick cultivation cycle makes it impossible to examine the complete plant life cycle. The minirhizotron method is an old in situ root research technique that uses dirt as a culture medium; however, it has drawbacks such as high costs, small in situ root pictures, and inefficient collection [13,14]. In contrast, methods such as x-ray computed tomography [15], magnetic resonance imaging [16], ground-penetrating radar [17], and electrical capacitance [18] have also been reported, but they are plagued by low imsenescence quality [1]. In contrast to earlier methods, the digital imsenescence device method [19,20] uses a high-resolution image acquisition device that has been widely used for root acquisition and offers the advantages of being less expensive and easier to install and demonstrating imsenescence. To get in situ root images of crops throughout their whole growth period, which can be used for studies on root senescence, this study makes use of the RhizoPot, which the team developed in the early stages [21,22].

Root identification is an approach to root phenotypic research, and traditional recognition methods include manual delineation and semiautomatic interactive recognition. The manual representation suffers from issues including low efficiency, a heavy workload, and a high rate of result error [23]. The researcher’s visual observations serve as the foundation for the root semiautomatic interactive software, and the auxiliary software identifies the roots, which can increase job productivity and lower labor expenses. However, relying on the arbitrary skill and practical knowledge of manual root separation makes it challenging to achieve high-throughput in situ root image recognition [24,25].

A deep-learning-based semantic segmentation approach speeds root identification research [26]. In addition, by continually extracting the features from the image’s region of interest, this technique achieves pixel-by-pixel categorization of the image. Since it was the first semantic segmentation model, fully convolutional networks (FCN) has produced positive results [27]. On the basis of this approach, Kamal et al. [28] accomplished weed-crop segmentation. The processing results for FCN are not fine enough, and the relationship between the pixels is not taken into account. To achieve end-to-end training with superior training results than FCN, SegNet utilizes a symmetric encoder–decoder structure based on FCN and adds nonlinear upsampling [29]. SegRoot, a high-throughput root analysis tool created by Wang and colleagues [30] that distinguishes between the soil and the root system, is based on the SegNet model. Originally developed as a segmentation model in the same year, UNet was first applied to the recognition of medical images. With skip connection structures added between levels, UNet is a U-shaped structure based on an encoder–decoder that integrates low-level characteristics and high-level semantic information to increase segmentation accuracy and make it more appropriate for small datasets [31]. As vascular tissue and the crop root system architecture are comparable, the model was applied to root identification. For instance, better UNet models were used to build root detection tools like ChronoRoot, RootPainter, RootDetector, etc. [32–34]. Pyramid pooling, on which PSPNet is built, aggregates the context data of many receptive fields and enhances the ability to gather global information [35]. On the basis of enhanced PSPNet, Zhang and colleagues [36] implemented agricultural regional segmentation. To reduce model parameters and increase accuracy, Google’s DeepLabV3plus model leverages Depthwise separable convolution and includes an encoder and decoder structure based on V3 [37]. In an earlier study conducted by our team, the enhancement of upsampling based on the V3plus network was documented [38,39]. Convolutional neural networks are the foundation for root feature extraction currently, but deeper networks, more input parameters, and more computation are needed to optimize and improve semantic segmentation models [40,41].

Li et al. [42], through improvements to UNet, achieved a pixel accuracy of 0.9917, an Intersection over Union (IoU) of 0.9548, and an F1 score of 95.10 in the task of peanut root recognition. Lu et al. [43] achieved a pixel accuracy of 97.7 in segmenting chili pepper roots, with an average F1 score over 90. Shen et al. [38], by enhancing deeplabV3plus for cotton root segmentation, reported F1 score, recall, and precision values of 0.9773, 0.9847, and 0.9702, respectively. Compared with the above papers, this study compares the 2 types of root segmentation models, the data used are from real root images with the same configuration environment, and the experimental results obtained were relatively accurate.

The machine translation issue was addressed in the initial proposal for the attention mechanism [44]. To process Natural Language Processing (NLP), the Google team created a transformer neural network that is entirely based on the attention mechanism block and uses the multihead self-attention mechanism in place of convolution [45]. The long-distance dependence issue is resolved by the transformer neural model, which is capable of performing parallel computations. Since the structure of the NLP input data is different from the structure of the picture data, it adopts a sequence structure. It has been published on how to apply transformer neural networks to the field of picture recognition [46]. This methodology achieves picture categorization by converting the image’s pixel and location data into a sequence via a patch operation. Alshammari et al. [47] used this model to implement the olive disease classification issue for plant disease detection. On the basis of this model, Chen et al. [48] finished classifying maize seeds. The vision transformer neural model can process images, but it has drawbacks, including a lot of parameters and calculations and a finite number of model stacks. Swin transformer (Swin) uses the window self-attention module and constructs a hierarchical structure to tackle the challenging problem of self-attention computing. Image data can now be transmitted between windows thanks to the shifted windows multihead self-attention structure [49], broadening the range of applications for transformer, including the detection of plant diseases, the identification of weeds, and the detection and identification of animals [50–53].

The visual attention mechanism model has a stronger ability to capture global receptive fields, extract more contextual information, and have good modal fusion ability, which has been widely used in agriculture [54]. Morphological information about color differences within plant tissues can be extracted based on red–green–blue (RGB) images [55], such as the analysis of sorghum senescence based on the degree of greenness in leaf tissues in the context of nitrogen and water availability [56], and the study of chlorophyll degradation during the senescence process of wheat and madder [57]. Typically, changes in root color indicate the degree of root senescence, and when the root turns black, it is thought to be dead [58]. There are not many reports on deep-learning-based root senescence recognition. To accurately identify in situ roots, the goal of this paper is to create a root segmentation model based on a visual attention mechanism. To assess this model’s processing effectiveness, we compare it to 2 widely used models: the convolutional model and the transformer model. The tracking and extraction of time-series root image characteristics are also lacking in root phenotypic investigations, and the present dynamic root identification is manually extracted by software during the root growth phase [59], which has issues with high cost and poor accuracy. In this study, full-fertility root pictures were precisely identified using this model by applying varying levels of nitrogen stress, which offers fresh perspectives for the investigation of crop time-series root dynamics properties.

## Materials and Methods

**Fig. 1.**
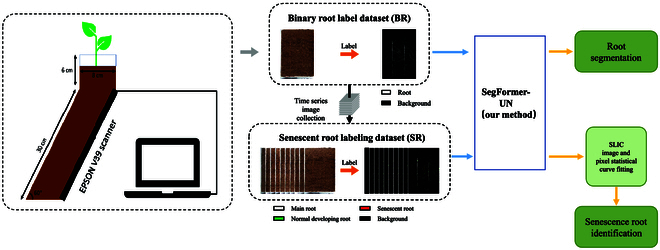
The overall process of dataset acquisition and model training.

The Crop Growth Regulation Laboratory at Hebei Agricultural University (Baoding City, Hebei Province, 38.85°N, 115.30°E) has an artificial climate chamber where this experiment was conducted. Figure 1 depicts the proposed method’s flow. To begin with, the root system time-series photos are obtained, and the dataset is created and tagged with 2 data kinds. Data type 1 is root binary annotation, and data type 2 is root senescence annotation. Subsequently, the improved model SegFormer-UN proposed in this paper is implemented for root segmentation training based on data type 1, and, finally, the senescent root recognition training is implemented based on data type 2.

### Data acquisition

**Fig. 2.**
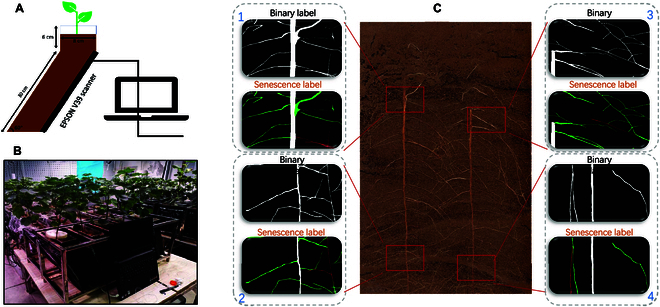
Image acquisition device and annotation method. (A) Image acquisition device. (B) Cluster acquisition. (C) Two annotation methods.

In situ root image acquisition was acquired using a high-throughput in situ root research device (RhizoPot) (Fig. 2A), which consists of a root’s growth device, and a flatbed scanner (Epson Perfection Version 39, Suwa, Japan) and a laptop. The root growth device is a trapezoid-like device composed of acrylic plates and attached to a scanner. The front of the device is 20 cm in width and 30 cm in height, the side is 8 cm in width, and the top protrusion is 6 cm in height. The growth device and scanner are at an angle of 60° to the ground and covered with a black straight plate to prevent light exposure. The flatbed scanner was connected to a PC. In addition, multiple scanner devices could be connected on the PC side through Epson’s dedicated Application Programming Interface (API) (Fig. 2B), and the final output image of the scanning device was in JPEG format.

As the object of study, the cotton plants were cultivated in 2 levels of nitrogen concentration soil: low nitrogen soil environment, 0 g N/kg^−1^; normal soil environment (urea N fertilizer), 138 mg N/kg^−1^. In addition, soil (superphosphate) (138 mg N/kg^−1^) and soil (potassium chloride) (138 mg N/kg^−1^) were applied to all soils. The greenhouse was maintained under the following environmental conditions: 14/10 h (day/night), 28°/25°C (day/night), a daytime light intensity of 600 μmol·m^−2^·s^−1^, and a relative humidity of 45% to 50%. Other cultivation conditions are as shown in the paper of Zhu et al. [60].

Image acquisition involved continuous capturing over a period of 100 d from the beginning of the crop growth stage. The pixel size of the image acquisition was 1,200 dpi (317.5 mm), with a resolution of 10,200 × 14,039 and an RGB image depth of 24 bits. The images were formatted as JPEG. There are 8 sets of root images, each consisting of over 100 images. Because of the unreliable factors during the scanning process, images containing noise and unclear content were excluded, limiting each set to 100 images. To complete the training of the model, a hundred images were randomly selected from the 8 sets for annotation. The selected images were divided into training sets, validation sets, and test sets with proportions of 70%, 20%, and 10%, respectively. The remaining images were used to assess the actual performance of the model. Adobe Photoshop CC 2020 (Adobe Inc., San Jose, CA, USA) was used for image annotation. The annotation process involved opening the images with Adobe Photoshop, creating a new layer, using the lasso tool to select the roots for annotation, and filling the selected roots with white using the paint bucket tool. This process was repeated until all root annotations were completed, and the soil background was filled with black. The annotation of 2 types of data is illustrated Fig. 2C. Four randomly selected localizations are shown, for example, diagrams of binary and senescence labeling.

#### Root binary annotation dataset

To finish training the model, the necessary dataset must be labeled. The root is symbolized in the labeled images by the color white, which has the color value [255, 255, 255]. The soil background is represented in black with a color value of [0, 0, 0]. The image is in PNG format. The actual annotated images, as shown in the binary label, took approximately 3 h for annotation per image. Because of the high resolution of the images in the training set, the original images are cropped into smaller images using windows of size of 768 × 768 dpi. In cases where the original image size is less than 768 × 768, black pixels are used to fill the gaps. The cropped real images and masks can fit completely after cropping. After cropping 100 large images, a total of 18,883 smaller images are obtained. These are divided into 3 datasets using a ratio of 70%, 20%, and 10%, resulting in a training set of 13,216 images, a validation set of 3,778 images, and a test set of 1,889 images. During model training, to ensure compatibility with the input requirements of the model, the images are resized to 512 × 512 dpi during the image reading stage.

#### Root senescence annotation dataset

To complete the labeling of the root senescence and reduce the labeling time, professional agronomy experts classify actual root systems based on binary images. The labeling categories are divided into 4 categories: Soil as the background is represented in black with the color value of [0, 0, 0]; normally growing root systems are represented in green with the color value of [0, 255, 0]; senescent roots are represented in red with the color value of [255, 0, 0]; and the main roots are represented in white with the color value of [255, 255, 255]. The annotation of aging roots requires approximately 1 h per image. Similarly, to manage the large image size, the entire image is cropped into smaller images with a size of 768 × 768 dpi. After cropping, the training set consists of 2,088 images, the validation set has 300 images, and the test set has 600 images. In addition, the images are resized to 512 × 512 dpi during the reading stage.

### Model structure

**Fig. 3.**
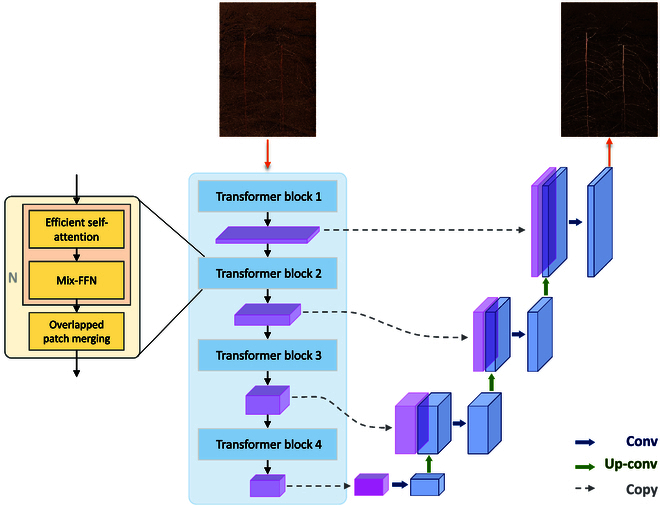
The overall structure diagram of the model.

This paper utilizes a U-shaped encoder and decoder structure and is based on the SegFormer model [61]. The encoder structure starts by extracting features through 4 transformer blocks, halving the size of the feature map after each extraction, and saving the output feature map of each block for feature fusion in the decoder. Both the encoder and decoder have 4 layers and are symmetrical. The decoding process entails stitching the transformer block’s output feature map, fusing it using convolution, and then upsampling to finish downscaling and dimensionalizing the image. The pixel-by-pixel classification of the image is finally finished by the segmentation head, and Fig. 3 depicts the model’s overall structure. This model uses 2 distinct backbones (small and large), whose depth varies and whose model layers are deeper for large than for small.

#### The transformer block of the encoder

In this study, the overlapped patch merging method is used to process the Vision Transformer (VIT)’s patch merging method, which can guarantee the local continuity of these patches at each stage of feature extraction. The patch interacts during feature extraction by varying patch size (*K*), stride between 2 adjacent patches (*S*), and padding size (*P*). Setting *K* = 7, *S* = 4, and *P* = 3 for the initial picture input and *K* = 3, *S* = 2, and *P* = 1 for the subsequent phases will complete this operation. The above operation enables to obtain a convolution-like multiscale output map in different feature processing stages after a given input image, assuming that the pixel size of the input image is *H* × *W* × 3 and the size of the feature map at different stages is as shown in Eq. 1:

$$\frac{H}{2^{i+1}}\times \frac{W}{2^{i+1}}\times C_{i}\;i\in \left\{1,2,3,4\right\}\;$$ (1)

where *C* in the formula denotes the image dimension of each stage.

The original single attention (SA) module is 3 times the sequence and input into SA (*Q*, *K*, *V*) for calculation, *Q* = *ZW_Q_*, *K* = *ZW_K_*, *V* = *ZW_V_*, and *W_Q_ W_K_ W_V_* ∈ *ℝ*^*C*×*d*^, where *Q*/*K*/*V* represents the weight matrix, *L* represents sequence length, *C* represents hidden channel size, and *d* represents output channel size. The final SA calculation is shown in Eq. 2:

SA=softmaxZWQZWKTdKZWV=softmaxQKTdKV (2)

where *d_K_* is the dimension of *K*, which is to prevent the inner product from being too large.

To improve the calculation efficiency, the calculation is simplified by reducing the *K* series, such as Eqs. 3 and 4:

$$\hat{K}=\text{Reshape}\left(\frac{L}{R},d\cdot R\right)\left(K\right)$$ (3)

$$K=\text{Linear}\left(d\cdot R,d\right)\left(\hat{K}\right)$$ (4)

where *R* represents the reduction ratio and $\hat{K}$ is to reshape the dimension to $\frac{L}{R}\times \left(d\cdot R\right)$ and then through Linear, $\hat{K}$ is reduced from *d* · *R* dimension to *d* dimension and, finally, $K\in {\mathbb{R}}^{\frac{L}{R}\times C}.$

Mix-FFN reduces the nonlinearity of the function by mixing the feed-forward network (FFN) after the efficient self-attention calculation. The calculation formula is such as Eq. 5:

$$x_{\text{out}}=\mathrm{MLP}\left(\text{GELU}\left({\text{Dconv}}_{3\times 3}\left(\mathrm{MLP}\left(x_{\text{in}}\right)\right)\right)\right)+x_{\text{in}}$$ (5)

where *x*_in_ is the input feature map, Dconv_3×3_ is the depthwise convolutions with convolution kernel 3, GELU is the activation function, and MLP is the multilayer perceptron.

#### Convolution of decoder

Convolution is mainly used to complete the segmentation operation in the decoder. The decoder used in this paper is similar to UNet, which recovers through convolution operations at different scales. Each layer of the decoder is mainly composed of upsampling and convolutional layers for feature map fusion and image size restoration. Finally, the segmentation head is used to achieve pixel by pixel classification to predict the actual root system.

### Extraction method of senescence root

Tasks involving senescence root extraction can be completed using the SegFormer-UN (small) suggested in this page. The senescence time series root dataset is used to train the model, which converges after 100 generations of training and infers the properties of the senescence root time series. This paper uses the superpixel approach (SLIC) for error correction because the shooting environment has an impact on the results of recognition. Senescence images are segmented into pixel blocks using the SLIC method, resulting in blocks of varying forms. To consistently cover nonroot pixels and protect the root block segmentation outcomes, a black and white prediction image is utilized. Each pixel block’s color weight value is calculated, and the color with a higher weight is used to replace the original color block. Calculate the senescence root and normal root pixels of each image once the temporal dataset has been repaired. Then, fit curves to the 2-pixel categories and contrast the results with the actual values. The algorithm is as follows.


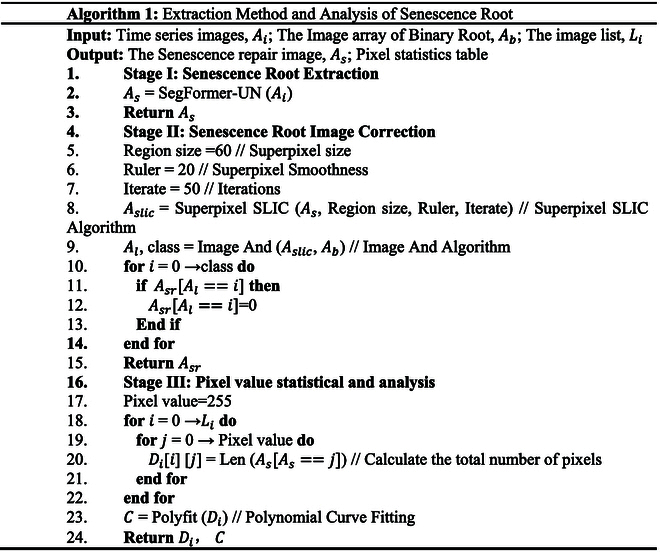


Among them, *A_s_* represents the senescence root matrix, *A_s_* represents the SLIC segmentation matrix, class represents the SLIC segmentation category, *A_l_* represents the image and algorithm output results, *A_sr_* represents the correction matrix, *D_i_* pixel statistical results, and *C* represents the fitting curve results.

### Experimental setup

To compare various semantic segmentation algorithms, a total of 7 models are selected on the basis of the usability and reproducibility of the code. The 4 convolutional neural networks are UNet, SegNet, PSPNet, and DeepLabV3plus, and transformer neural networks are TransUNet, SwinUNet, and SETR [62–64].

The training and inference of all the above models are based on the Ubuntu 22.10 Operating System, and the configuration of the system is as follows: The processor is Intel i5-12400F (2.5GHz) with 32-GB RAM, the graphics card model is NVIDIA RTX 3060 with 12-GB graphics memory, and a memory bit width of 192 bits. The deep learning framework used for training is PyTorch 11.6.

All models are trained in the same training environment with the same model hyperparameter values to ensure fairness during training. The parameters betas are set to 0.9 and 0.999, the initial learning rate is set to 0.0001, and the learning rate decay strategy uses the cosine annealing learning rate decay strategy in all of the models. They also all adopt the adaptive moment estimation with decoupled weight decay (adamW) optimizer (CosineAnnealingLR). Moreover, the model training spans 100 generations. The appropriate data enhancement is done during the training phase to guarantee the training effect and boost the robustness and generalizability of the model. The common techniques for data augmentation include flipping, cropping, and altering image properties (brightness, contrast, saturation, and hue).

### Evaluation of the model

To validate various model performances, the model needs to be evaluated with the help of evaluation metrics. In this paper, the root system and soil background are equivalent to the pixel-by-pixel classification of images, so it is necessary to use the confusion matrix to count the classification results and actual values to further obtain a variety of classification basis. In this paper, 4 evaluation metrics are Precision (Eq. 6), Recall (Eq. 7), and IoU (Eq. 8), and F1 score (Eq. 9), which are calculated as shown below:

$$\text{Precision}=\frac{\text{TP}}{\text{TP}+\text{FP}}\times 100\%$$ (6)

$$\text{Recall}=\frac{\text{TP}}{\text{TP}+\text{FN}}\times 100\%$$ (7)

$$\text{IoU}=\frac{\text{TP}}{\text{TP}+\text{FN}+\text{FP}}\times 100\%$$ (8)

$$F1=2\times \text{Precision}\times \frac{\text{Recall}}{\text{Precision}+\text{Recall}}\times 100\%$$ (9)

In the formula, TP stands for the number of root pixels that were accurately predicted to be roots, FP is for the number of background pixels that were accurately predicted to be roots, FN is for the number of background pixels that were correctly predicted to be roots, and TN is for the number of background pixels that were accurately predicted to be backgrounds. IoU can assess how closely classification results for pixels match actual values. F1 is the harmonic average of Precision and Recall, which are used to confirm that the rate of pixel classification is correct. Each of the 4 evaluation criteria has a value range of 0 to 1.

## Results

### Segmentation results

This paper compares a total of 8 models, including PSPNet, SegNet, UNet, DeeplabV3plus, TransUNet, SwinUNet, SETR, and the method proposed in this paper. Each model is trained using the same configuration for 100 epochs, and all model losses reach convergence, and the model training effect is tested on the basis of the test set to obtain the evaluation indexes. Considering the indicators and segmentation results comprehensively, the optimal model is selected. The numerical comparison results of the model indicators are shown in Table 1. The SegFormer-UN (small) proposed in this paper uses a lightweight backbone with relatively small Floating-point Operations (FLOPs) and Params. The mIoU and mRecall indicators are also higher than other comparative models at 81.06% and 86.29%, respectively. Since mPrecision and mRecall are a pair of contradictory values, mF1 is used to measure the quality of both. The mF1 value of SegFormer-UN (small) is 88.47%. The best evaluation indicators are obtained when a deeper backbone model SegFormer-UN (large) is used, with the largest values of mIoU, mRecall, and mF1, which are 81.52%, 86.87%, and 88.81%, respectively. The deeper backbone increases FLOPs and Params but still lower than that of the other transformer neural networks.

**Table. 1.** Evaluation metrics of the model

Method	Flop (M)	Params (M)	Root IoU (%)	Root Recall (%)	Root Precision (%)	F1 (%)
UNent	**786,318.75**	**34.53**	**80.87%**	**85.46%**	**91.75%**	**88.33%**
DeepLabV3plus	**95,122.49**	**22.44**	**78.03%**	**82.07%**	**91.49%**	**86.14%**
PSPnet	**28,367.25**	**1.51**	**76.43%**	**79.61%**	**92.49%**	**84.83%**
SegNet	**937,999.47**	**39.79**	**79.79%**	**85.27%**	**90.09%**	**87.52%**
SETR	**275,440.73**	**85.88**	**75.86%**	**81.07%**	**88.53%**	**84.37%**
TransUNet	**351,034.49**	**91.52**	**79.53%**	**84.33%**	**90.92%**	**87.31%**
SwinUNet	**136,384.82**	**41.34**	**79.99%**	**85.07%**	**90.73%**	**87.67%**
SegFormer-UN (small)	**15,528.10**	**5.81**	**81.06%**	**86.29%**	**90.96%**	**88.47%**
SegFormer-UN (large)	**60,972.07**	**23.11**	**81.52%**	**86.87%**	**90.98%**	**88.81%**

**Fig. 4.**
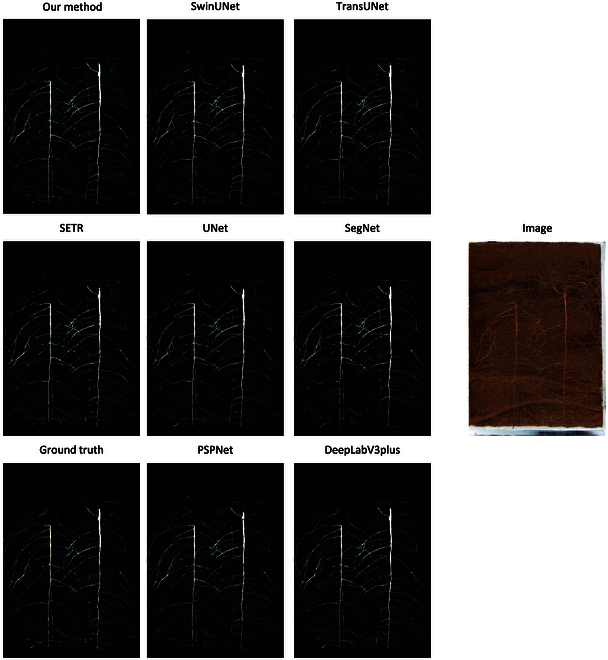
Root segmentation image.

The root segmentation results are shown in the Fig. 4. Among the aforementioned models, except for SETR, all segmentation models exhibit excellent results in root segmentation. The segmentation of root edges is smooth, and overall recognition is clear, although there are some differences in certain details. However, the SETR model shows more spikiness in the segmentation of root edges, and there are instances of interrupted root recognition.

**Fig. 5.**
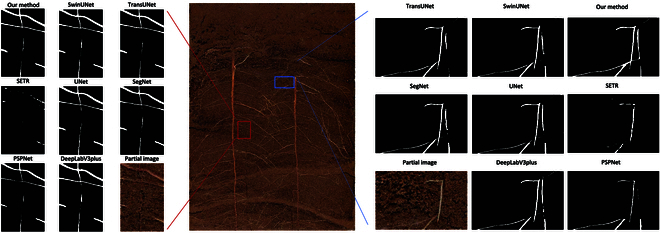
Root segmentation results.

Our improved method handles the boundaries of root systems more effectively (as indicated by the blue box in Fig. 5). SegFormer-UN can recognize and connect the low-contrast roots and recognize more and more complete roots, while other models exhibit discontinuities in root recognition, and some models are unable to identify certain roots. However, when dealing with the issue of significant soil particle occlusion, the results of all the aforementioned models are not ideal (as highlighted by the red box in Fig. 4). Because of severe soil particle occlusion, some roots are in a blurred and indistinct state, making it challenging for all segmentation models to accurately identify the roots. However, the model proposed in this study, along with SwinUNet and TransUNet, performs relatively better compared to other models in segmenting obscured roots.

To further validate the effectiveness of the improved model, we conduct ablation experiments on a series of methods adopted in this paper, and the comparison results are shown in Table 2. The experimental methods include improvement of 2 upsampling methods subpixel (SegFormer-Sub-pixel) and deconvolution (SegFormer-Trans-Conv), UNet decoder (SegFormer-UN), Depthwise separable convolution+UNet decoder (SegFormer-DU), and DeeplabV3plus decoder (SegFormer-DP). The results show that the model structure using the improved upsampling method alone has decreased compared to the original model evaluation indicators. Among them, the mIoU, mRecall, mPrecision, and mF1 of SegFormer-Trans-Conv have decreased by 79.09%, 84.17%, 90.34%, and 86.98%, respectively; the mIoU, mRecall, mPrecision, and mF1 of SegFormer-Sub-pixel decreased to 78.99%, 84.02%, 90.37%, and 86.90%, respectively. The performance of the model varies after changing the decoder. The SegFormer-DP indicator value decreases, and the required Params also increases. The SegFormer-UN and SegFormer-DU indicators have both improved and reduced model FLOPs and Params. Among them, SegFormer-UN has the greatest improvement, with 4 indicators of 81.52%, 86.87%, 90.98%, and 88.81%, and the FLOPs and Params of model have been slightly reduced. As we continue to increase the depth of the model backbone, the improvement effect becomes more obvious. The 4 indicator values increase by 1.34%, 1.49%, 0.34%, and 0.99%, respectively, compared to the original model, but these will be accompanied by a doubling of FLOPs and Params.

**Table. 2.** Evaluation metrics of the model

Method	Flop (M)	Params (M)	Root IoU (%)	Root Recall (%)	Root Precision (%)	F1 (%)
SegFormer (small)	**125,096.17**	**6.08**	**80.18** **%**	**85.38** **%**	**90.64** **%**	**87.82** **%**
SegFormer-Trans-Conv (small)	**135,087.00**	**6.28**	**79.09** **%**	**84.17** **%**	**90.34** **%**	**86.98** **%**
SegFormer-Sub-pixel (small)	**125,096.17**	**6.08**	**78.99%**	**84.02%**	**90.37%**	**86.90%**
SegFormer-DP (small)	**38,425.59**	**7.81**	**79.54%**	**84.57%**	**90.61%**	**87.33%**
SegFormer-DU (small)	**10,218.50**	**3.93**	**80.92%**	**86.34%**	**90.66%**	**88.37%**
SegFormer-UN (small)	**15,528.10**	**5.81**	**81.06%**	**86.29%**	**90.96%**	**88.47%**
SegFormer-UN (large)	**60,972.07**	**23.11**	**81.52%**	**86.87%**	**90.98%**	**88.81%**

### Extraction of time-series root senescent features

**Fig. 6.**
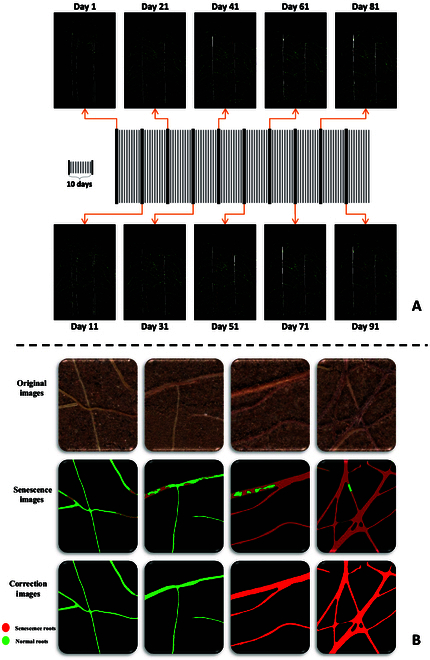
(A) Time series senescence root results. (B) Senescence correction results.

Senescence root color weights can be calculated automatically on the basis of annotated images using the SegFormer-UN model, which leads to better root extraction performance (processing time and image recognition) than image processing. On the basis of deep learning extraction methods, graphics processing unit (GPU) can be used to accelerate inference operations, completing a 512 × 512 dpi image inference takes about 1 s, and completing a complete image takes about 4 min. It is possible to notice that root senescence is increasing using an improved model to extract the sequence senescence root dataset with 10-d intervals, as illustrated in Fig. 6A. Similar to this, although deep learning can swiftly extract roots, there still exist senescent and normal root systems in the same root system in a mixed state. The mixed colors will entirely be replaced by colors with large weight based on the superpixel correction results displayed in Fig. 6B. Using superpixel correction and conducting manual recognition for comparison, it was found that the accuracy of aging root system identification relatively improved after correction. Small blocks of misclassified roots are corrected without affecting the overall root system classification. Therefore, from a pixel perspective, the corrected identification results for aging root systems are more accurate. The training and inference configurations are the same as those for root segmentation.

### Exploration of time series root senescence law

**Fig. 7.**
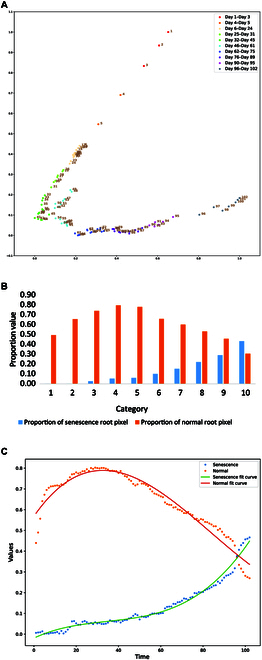
(A) Time series senescence cluster analysis results. (B) Pixel ratio histogram of normal and senescence root at time nodes. (C) Pixel fitting curves for normal and senescence root.

Because of the similarity of adjacent images in temporal data, dimensionality reduction and clustering are used to find the maximum difference between images and to find the proportion of normal and senescence root pixels under temporal and statistical nodes (Fig. 7A and B). The clustering results prove that the temporal dataset is classified into 10-time intervals with different time intervals according to the growth order. The proportion of senescence root in the initial growth stage is zero, and the proportion of senescence root increases with time. However, compared to normal root, the proportion of senescence root is not significant, and the trend of senescence root in the later stage of root growth is increasing. The trend of senescence root changes over time is consistent with the observation results of the naked eye. For the normal root, the percentage of the normal root system increases because of the early stage of being in the growth phase with vigorous root growth, and the total root grows slowly in the middle and late stages, and the true ratio of the normal root decreases accordingly after the senescent root system increased. This paper performs cubic polynomial fitting on the proportion of normal and senescence pixels. The fitting results are shown in the figure, and the 2 curves have good fitting effects. The *R*^2^ of the senescence fitting curve is 0.98, and the mean squared error is 0.00025. The *R*^2^ of the normal fitting curve is 0.94, and the mean squared error is 0.00113.

## Discussion

### Data annotation logic

We use 2 forms of labeling rather than unique senescent labeling to label the data due to labeling expense and training issues. First of all, when compared to a senescent label, semantic segmentation reduces labeling time by 2 h, and integrating 2 datasets can cut total labeling time. There is not a strict restriction for the number of senescent labels in the dataset because the data we gather consists of serialized images with a high degree of similarity. Second, we merely use the black box model of deep learning in our senescent extraction to simply understand the color difference of the root system.

### Model analysis

The findings of the model comparison show that the transformer neural network’s index evaluation is superior to that of the convolutional neural network. This is a result of the self-attention mechanism’s capacity for direct global relationship modeling, which increases the image’s receptive field and gathers additional contextual data. However, in terms of processing and parameters, a transformer neural network frequently exceeds a convolutional neural network. The SegFormer model reported in this research improves the self-attention mechanism, producing the smallest SegFormer (small) model with fewer parameter values and better performance than traditional convolutional neural networks. However, the details on the root of the transformer neural network are more detailed, which is also a result of the self-attention mechanism’s capacity to process global information. The processing results of the transformer neural network and the convolutional neural network in terms of actual image segmentation do not differ significantly.

Although the output results of the model are not significantly different from the rest of the convolution and transformer, the detailed recovery of the feature map is still insufficient. The original model decoding method is the output feature map of the 4 transformer blocks, using a straightforward and lightweight MLP plus sampling method for feature stitching fusion output results.

The initial approach in this paper was improved using 2 different upsampling methods (subpixel and deconvolution) in light of earlier studies by this experimental group, and it is evident from the experimental results that the above 2 methods are ineffective for model enhancement. This is due to the fact that adjusting the upsampling alone is insufficient to restore the target, there is an unavoidable loss of details in the preceding procedures, and the contextual information from the backbone extraction is not effectively used.

As target information and dimensions are restored, this paper adopts a tweak in the decoder structure to minimize detail loss. This paper uses the DeeplabV3plus decoder structure initially but does a bad job of utilizing scale feature maps, which leads to subpar processing results for root. While gradual convolution and feature stitching improve semantic segmentation, we adopted the UNet decoder structure for feature restoration. By using a skip connection to mix data from various trunk stages throughout the upsampling recovery process, low-level detail data and high-level semantic data may be recovered and combined more effectively, improving segmentation accuracy. After improvement, we discovered that the model’s computational complexity was lowered by an eighth compared to the original decoding structure, and the number of parameters was decreased by around 0.27 million, shortening training time. This is owing to the original model’s excessive usage of hidden layers and the MLP structure, which also causes the loss of spatial information in the feature map, resulting in a huge number of parameters.

To further simplify the model, the use of Depthwise separable convolution instead of convolution further reduces the number of parameters and computational complexity. Depthwise separable convolution first uses deep convolution to perform spatial convolution on each channel of the feature map and then uses point-by-point convolution to fuse the channel information, which simplifies the convolution operation through step-by-step operations. Although the number of parameters is reduced compared to the convolutional method, the evaluation indicators are not as good as those of the convolutional indicators.

The SegFormer-UN training results achieved were the best of all models, and in addition to making improvements to the decoder, we also attempted to enhance the depth of the backbone, which entails an increase in computation and parameter count. Although the model’s parameter count has increased considerably compared to SegFormer (small), SwinUNet, and TransUNet and there are certain advantages, the computation increase is not considerable.

### Senescent root extraction

#### Traditional methods

**Fig. 8.**
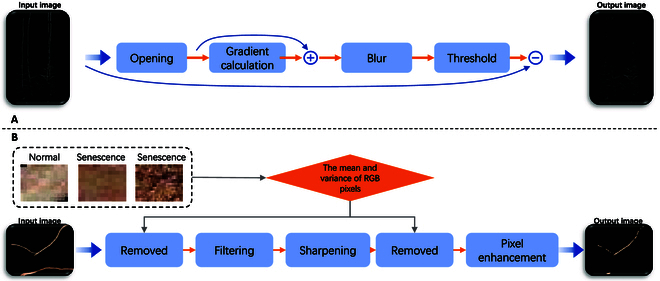
(A) Flowchart for removing the taproot. (B) Flowchart for extracting senescence root.

Before classification, the taproot must be removed because its existence will prevent correctly extracting senescent roots from the root segmentation image after it has been obtained [65]. To build the full root, first, remove the majority of the vertical taproot from the predicted image using the opening operation, then calculate the root edge using the image gradient, enlarge it using blur and threshold, and finally combine it with the opening operation results. Last, lateral root extraction is achieved by images and operations. The categorization root pixel block is then intentionally picked to determine the mean and variance of the pixels (one normal root and 2 senescent roots). The senescent root pixels are filtered by mean and variance to retain the normal root pixels, and the retained results are filtered and sharpened. The senescent root pixel is then removed once again by means and variance, and both the final filtered image and the original image are pixel enhanced. If the calculated value is greater than 0, the original image pixel block is assigned to the filtered image until the sliding window is finished. Pixel enhancement involves calculating the 2-norm difference between the 2 image pixel blocks in accordance with the sliding window with the initial pixel core size of 2 and the maximum pixel size of 4 [66]. Figure 8 depicts the image processing procedure.

Mean and variance of RGB channel pixels value are easily determined by artificially creating 3 separate pixel blocks, and the outcome of removing the senescent root is acquired after pixel-by-pixel processing. The lower, darker root system has been eliminated, leaving only the top, light-colored live root visible in the image. While some pixel values around the classification threshold are deleted from the local result map, the upper portion of the normal root of the complete root system is mistakenly left behind. Senescent root and normal root are now separated on the same root; however, this is an atypical occurrence. The computation time for extraction is too long compared to deep learning because this method cannot be computed using GPU; it takes roughly 31 min to calculate one image, and this time rises when dealing with dense distributions of roots.

#### SegFormer-UN

At present, color classification based on deep learning is widely used in different fields and has high practicability. For instance, Yang et al. [67] classified feces using deep learning to extract shallow information in the medical industry. Liu et al. [68] successfully identified the apple root system in agriculture using various seedling root colors. As a result, the strategy described in this work, which is based on deep learning, is effective. The modified model for root senescent extraction has a high extraction effectiveness in the inference stage according to experiments, and it extracts data quickly. This is due to the trained model’s ability to automatically determine the senescent root’s classification threshold and utilize GPU acceleration to speed up processing. The use of the SegFormer (small) model in the inference stage is characterized by short processing time and high extraction efficiency. Considering the application of this method to mobile devices, a relatively smaller model is chosen. Compared to image processing, the overall training samples are small, and the cost is relatively low, although the model costs extra during training. Zhu et al. [69] showed that manually tracking and extracting the senescence root from time series information takes a lot of time. This study improves efficiency by achieving one-stop, quick identification and analysis with deep learning and image processing.

More image processing is required in this piece since deep learning approaches have an issue with intermittent error recognition. Then, we classify senescence photos into pixel blocks, which are further separated into blocks with various forms, using the superpixel approach. Then, we cover the image with binary prediction, keeping only the necessary root, and recolor the processing output to mostly remove false positives. Although the aforementioned method can fix a tiny percentage of wrong colors, it cannot fix vast mistake recognition areas. There are many errors in root senescence identification, including subjective annotation errors and the impact of model performance. However, compared to purely manual recognition, the efficiency improvement brought about by the model is beyond what can be achieved manually. Moreover, varied shooting environments might result in varying exposure, brightness, and contrast among temporal data photos, which can result in inaccurate root recognition as a result of the aforementioned factors.

## Conclusion

This paper uses transformer neural networks to recognize roots and extract senescence roots from temporal pictures. In comparison to transformer and generic convolutional neural networks, the SegFormer-UN model performs better and can accurately partition cotton roots. The best root mIoU, mRecall, and mF1 index values were obtained by the model, which were 81.52%, 86.87%,and 88.81%, respectively. It is more accurate to use the real root segmentation image connection. The updated picture based on SegFormer-UN was proven to be more precise and effective in root extraction than conventional image processing techniques after 2 senescence root extraction approaches were verified. However, we are unable to receive full acknowledgment. We will eventually broaden the variety of root senescence samples and enhance network performance. The senescence laws of cotton root can be investigated using the senescence root extraction technique created by our research center.

## Data Availability

The dataset could be given upon reasonable request from the corresponding author N.W.
